# Machine Learning-Based Prediction of Long-Term Mortality in Patients with Implantable Cardioverter Defibrillators

**DOI:** 10.3390/jcdd13100514

**Published:** 2026-10-12

**Authors:** Lei Pan, Zilong Xiao, Ziqing Yu, Siyuan Li, Weiwei Zhang, Ruogu Li, Zhongkai Wang, Hongyang Lu, Wei Hua, Yangang Su, Yixiu Liang

**Affiliations:** 1 Department of Cardiology, Zhongshan Hospital of Fudan University, Shanghai Institute of Cardiovascular Diseases, National Clinical Research Centre for Interventional Medicine, Shanghai 200032, China; panleiyaomingkaka@126.com (L.P.); xiao1453497595@163.com (Z.X.); yu.ziqing@zs-hospital.sh.cn (Z.Y.);; 2 Shanghai Medical College, Fudan University, Shanghai 200032, China; lisiyuan24@m.fudan.edu.cn; 3 Department of Cardiology, Shanghai Chest Hospital of Shanghai Jiao Tong University, Shanghai 200030, China; zww1230523@163.com (W.Z.); liruogu@hotmail.com (R.L.); 4 Department of Cardiology, Changhai Hospital, Naval Medical University, Shanghai 200433, China; 13816038626@163.com; 5 Cardiac Rhythm Management, Medtronic Technology Center, Medtronic (Shanghai) Ltd., Shanghai 201203, China; 6 State Key Laboratory of Cardiovascular Disease, Cardiac Arrhythmia Center, Fuwai Hospital, National Center for Cardiovascular Disease, Chinese Academy of Medical Sciences and Peking Union Medical College, Beijing 100037, China; drhuaweifw@sina.com

**Keywords:** machine learning, mortality, implantable cardioverter-defibrillators, prognosis

## Abstract

**Aims**: Accurate prediction of the clinical course in implantable cardioverter-defibrillator (ICD) and cardiac resynchronization therapy-defibrillator (CRT-D) patients is crucial, as their disease progression varies significantly due to comorbidities and individual characteristics. **Methods**: This retrospective study included 3321 patients from four hospitals in China, with 3207 for model development and internal validation and 114 for external testing. Five machine learning (ML) models were evaluated using the C-index, time-dependent AUROC, and Brier score. Decision curve analysis (DCA) assessed clinical utility, while Kaplan–Meier curves stratified patients into high-, intermediate-, and low-risk groups. SHAP analysis identified key predictors and explained model outputs. **Results**: The Random Survival Forest (RSF) model demonstrated favorable numerical performance across multiple evaluation metrics, achieving the highest time-dependent AUROC (0.74, 0.74) and C-index (0.71, 0.70) in internal and external test sets. It also had the lowest time-dependent Brier score (0.17) in the internal test and ranked second (0.13) in the external test. DCA demonstrated numerically higher net benefit for the RSF model across evaluated threshold probabilities. Kaplan–Meier curves revealed distinct survival trajectories, with high-risk patients showing significantly higher mortality than low-risk patients (HR: 4.50, 95% CI: 3.10–6.55, *p* < 0.0001), while intermediate-risk patients also had increased mortality (HR: 2.49, 95% CI: 1.69–3.68, *p* < 0.0001). LVEDD was the most influential predictor of mortality. **Conclusions**: The RSF survival model enables individualized, time-resolved prediction of all-cause mortality risk in ICD and CRT-D patients, supporting risk stratification and future evaluation of clinical utility, while requiring cautious interpretation of longer-term predictions due to limited numbers at risk.

## 1. Introduction

The widespread adoption of implantable cardioverter-defibrillators (ICDs) and cardiac resynchronization therapy defibrillators (CRT-Ds) in modern healthcare underscores the need for precise risk stratification [1,2]. Patients receiving these devices exhibit significant variability in disease progression due to comorbidities and individual characteristics [3]. Accurately identifying high-risk patients after device implantation is essential for guiding appropriate follow-up and optimizing post-implantation care.

Several predictive models have been developed to assess the risk of ICD/CRT-D patients, with varying focuses. Some models predict the occurrence of ventricular tachyarrhythmia [4,5,6,7], while others estimate mortality risk accounting for competing events [2,3,8,9,10,11]. Most models primarily aim to identify patients suitable for ICD implantation. However, most existing models provide only static risk estimates, failing to account for how risk evolves over time [12,13]. This limitation reduces their clinical utility, as understanding when a patient is at the highest risk is as crucial as knowing whether they are at risk at all.

In this study, we develop and evaluate machine learning (ML) models to predict all-cause mortality in ICD and CRT-D patients, shifting the focus from merely identifying candidates for device implantation to optimizing post-implantation management [8]. Unlike traditional models that provide static or binary risk estimates [12,13], our approach generates individualized, time-to-event survival probability estimates across a predefined 10-year prediction horizon. This enables a more flexible and precise understanding of patient prognosis, supporting timely clinical interventions. By leveraging this framework, we aim to enhance post-implantation risk stratification and support individualized follow-up planning and future strategies for optimizing patient management.

## 2. Methods

### 2.1. Study Population

**Figure 1:**
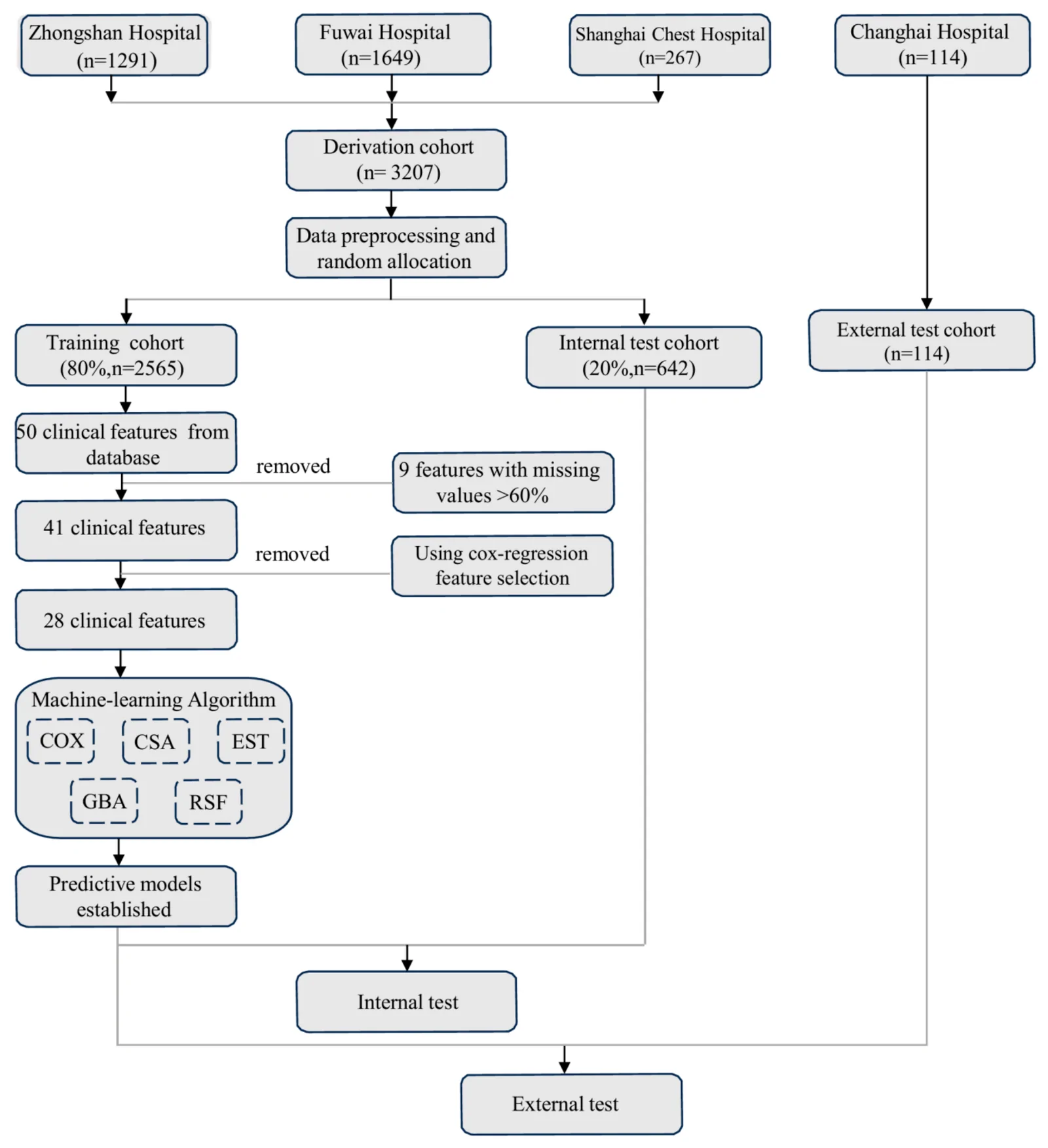
Flowchart illustrating the steps of patient selection, feature extraction, model training and testing. COX, Cox Proportional Hazards Model; CSA, Coxnet Survival Analysis Model; EST, Extra Survival Trees; GBA, Gradient Boosting Survival Analysis; RSF, Random Survival Forest.

Consecutive patients who underwent ICD or CRT-D implantation at 4 major implantation centers in China were retrospectively screened. Patients from 3 (Zhongshan Hospital from March 2004 to March 2021, Fuwai Hospital from January 1999 to October 2020, and Shanghai Chest Hospital from January 2015 to March 2022) of the 4 centers constituted the derivation set and were randomly divided into a training set (80%) and an internal test set (20%). All patients from the remaining center (Shanghai Changhai Hospital from July 2009 to August 2022) formed the external test set (Figure 1).

The study was approved by the ethics committee of Zhongshan Hospital (No. B2021-292R), and all patients provided written informed consent. The study adheres to the principles outlined in the Declaration of Helsinki and was registered in ClinicalTrials.gov (NCT05396313).

### 2.2. Data Collection and Pre-Processing

Clinical records within the first 24 h of admission were collected, covering medical history, biochemical tests, electrocardiographic and echocardiographic parameters, and baseline medication. Information on survival status was obtained from the Chinese household registration management system at the close of December 2022.

Initially, 50 clinical variables were considered as candidate predictors. Variables with more than 60% missingness were excluded before imputation, leaving 41 candidate predictors for feature selection. Dataset splitting was performed before feature selection and model development. Missing values in the remaining variables were imputed separately within the training, internal test, and external test cohorts using the IterativeImputer algorithm [14,15,16]. The missingness of candidate predictors across the training, internal test, and external test cohorts is summarized in Table S1. Afterward, continuous variables were standardized separately within the training, internal test, and external test cohorts, and categorical variables were converted into binary dummy variables through one-hot encoding.

### 2.3. Model Development

After data splitting, feature selection was performed exclusively within the training cohort before model development. Cox regression was used as a predictive feature selection framework to identify informative predictors for subsequent survival model development. Candidate predictors were initially ranked according to their predictive discrimination based on the Harrell’s C-index derived from univariable Cox models. Subsequently, SelectKBest, implemented in scikit-learn (version 1.6.1),was used to evaluate different numbers of retained predictors (k). During this process, feature ranking and selection were repeated within each fold of three-fold cross-validation in the training cohort, and the optimal number of retained predictors was determined according to the highest mean validation C-index from multivariable Cox models. After determining the optimal number of predictors, the feature-selection pipeline was refitted using the complete training cohort to obtain the final predictor set, which was fixed for subsequent model development and hyperparameter optimization. The internal and external test cohorts were not involved in any feature ranking, selection, or determination of the final predictor set and were reserved only for final model evaluation. The final list of 28 selected clinical predictors is provided in Table S2.

Using the selected predictors, five survival models were developed in the present study, including the Cox Proportional Hazards Model (COX) as a conventional statistical reference model, Coxnet Survival Analysis Model (CSA), Extra Survival Trees (EST), Gradient Boosting Survival Analysis (GBA), and Random Survival Forest (RSF) (Figure 1).

Hyperparameters were optimized exclusively within the training cohort using exhaustive grid search with five-fold cross-validation. The cross-validation folds were stratified according to mortality status and randomly shuffled with a fixed random seed. The mean Harrell’s C-index across validation folds was used as the optimization metric, and the optimal hyperparameter combination was subsequently used to refit the final models using the complete training cohort. The hyperparameter search spaces and best-tuned parameters for each model are provided in Table S3.

### 2.4. Model Evaluation

Model discrimination was measured using the concordance index (C-index). Due to the continuous nature of survival outcomes, the average area under the receiver operating characteristic curve (AUC) for each year was also calculated. Furthermore, each year’s average Brier score was calculated using the mean squared difference between observed and predicted outcomes to assess the accuracy of survival probability predictions. Calibration was assessed for the RSF model at 1-, 3-, and 5-year horizons using calibration plots comparing predicted mortality risk with Kaplan–Meier-estimated observed mortality risk. Calibration slope, calibration intercept, and calibration-in-the-large were calculated to quantify agreement between predicted and observed risks. Observed-to-predicted mortality risk ratios and observed versus predicted mortality risks across deciles of predicted risk were additionally evaluated. Decision curve analysis (DCA) was performed to evaluate the potential clinical utility of the models by quantifying net benefit across a range of threshold probabilities. In this study, threshold probabilities were interpreted as potential risk thresholds for identifying patients who may benefit from additional clinical evaluation or closer follow-up rather than as predefined treatment decision thresholds.

Subgroup analyses were also performed to evaluate model performance across primary vs. secondary ICD, ICD vs. CRT-D, and ischemic vs. nonischemic cardiomyopathy.

For comparison with an established clinical benchmark, the MADIT II score was used as a baseline model. The MADIT II integer score was recalibrated in our cohort using a Cox model to generate 5-year mortality probabilities, which were then directly compared with RSF predictions. Differences in discrimination were assessed using the C-index, and the net reclassification index (NRI) was calculated to evaluate the improvement in risk stratification provided by RSF relative to MADIT II.

### 2.5. Survival Analysis

After selecting the best model based on the highest C-index, mean AUROC, and lowest Brier score, patients were evenly classified into high-, intermediate-, and low-risk groups according to their ascending 5-year survival probabilities predicted by the model. Kaplan–Meier curves were generated to visualize survival differences among risk groups, and statistical significance was assessed using the log-rank test.

### 2.6. Interpretability Analysis

We used the Shapley additive explanations (SHAP) method to evaluate and prioritize the contribution of selected features to model predictions. SHAP values quantify the magnitude and direction of each feature’s contribution to the model output. Positive SHAP values indicate contributions toward higher predicted risk, whereas negative SHAP values indicate contributions toward lower predicted risk.

### 2.7. Statistical Analysis

Variables with a normal distribution were displayed as mean ± SD and assessed using Student’s *t*-test, whereas non-normally distributed variables were expressed as median (interquartile range) and evaluated with the Mann-Whitney U test. Categorical variables were displayed as counts and percentages and compared with the chi-square test. Kaplan–Meier survival curves were compared using log-rank tests. A two-sided *p* value of less than 0.05 was deemed statistically significant. All analyses utilized open-source Python (version 3.10, Python Software Foundation, Wilmington, DE, USA) and R software (version 3.6, R Foundation for Statistical Computing, Vienna, Austria).

## 3. Results

### 3.1. Baseline Characteristics

The derivation set comprised 1291 patients from Zhongshan Hospital, 1649 patients from Fuwai Hospital, and 267 patients from Shanghai Chest Hospital. The external test set consisted of 114 patients from Changhai Hospital. Thus, a total of 3321 patients receiving ICD or CRT-D implantation were included in the present study (Figure 1).

The baseline characteristics of the included patients are summarized in Table 1. The median age of all patients was 61.0 years (IQR 51.0, 68.0), with patients in the external test cohort being significantly older than those in the derivation cohort (70.02 years [IQR 63.01, 75.03] vs. 61.02 years [IQR 51.03, 68.01], *p* < 0.01). Compared to the derivation cohort, the external test cohort had significantly lower serum creatinine levels (30.39 [18.00, 75.01] vs. 87.01 [IQR 73.35, 103.02], *p* < 0.01). Additionally, significant differences were observed in serum sodium levels, N-terminal pro-B-type natriuretic peptide (NT-pro BNP) levels, left atrial diameter, and the use of key medications, including ACE inhibitors, ARNI, beta-blockers, and statins (*p* < 0.01).

During follow-up, all-cause mortality occurred in 848 (33.0%), 216 (33.7%), and 18 (15.8%) patients in the training, internal test, and external test cohorts, respectively. Median follow-up duration was 4.10 years (IQR, 2.34, 6.54), 3.99 years (IQR, 2.23, 6.31), and 3.15 years (IQR, 1.56, 4.90), respectively. The total person-years of follow-up and numbers at risk at 1, 3, 5, and 10 years are provided in Table S4.

Device characteristics and clinical indications, including ICD versus CRT-D implantation, primary versus secondary prevention, and ischemic versus nonischemic etiology, are summarized across the training, internal test, and external test cohorts in Table S5.

### 3.2. Model Performance

**Figure 2:**
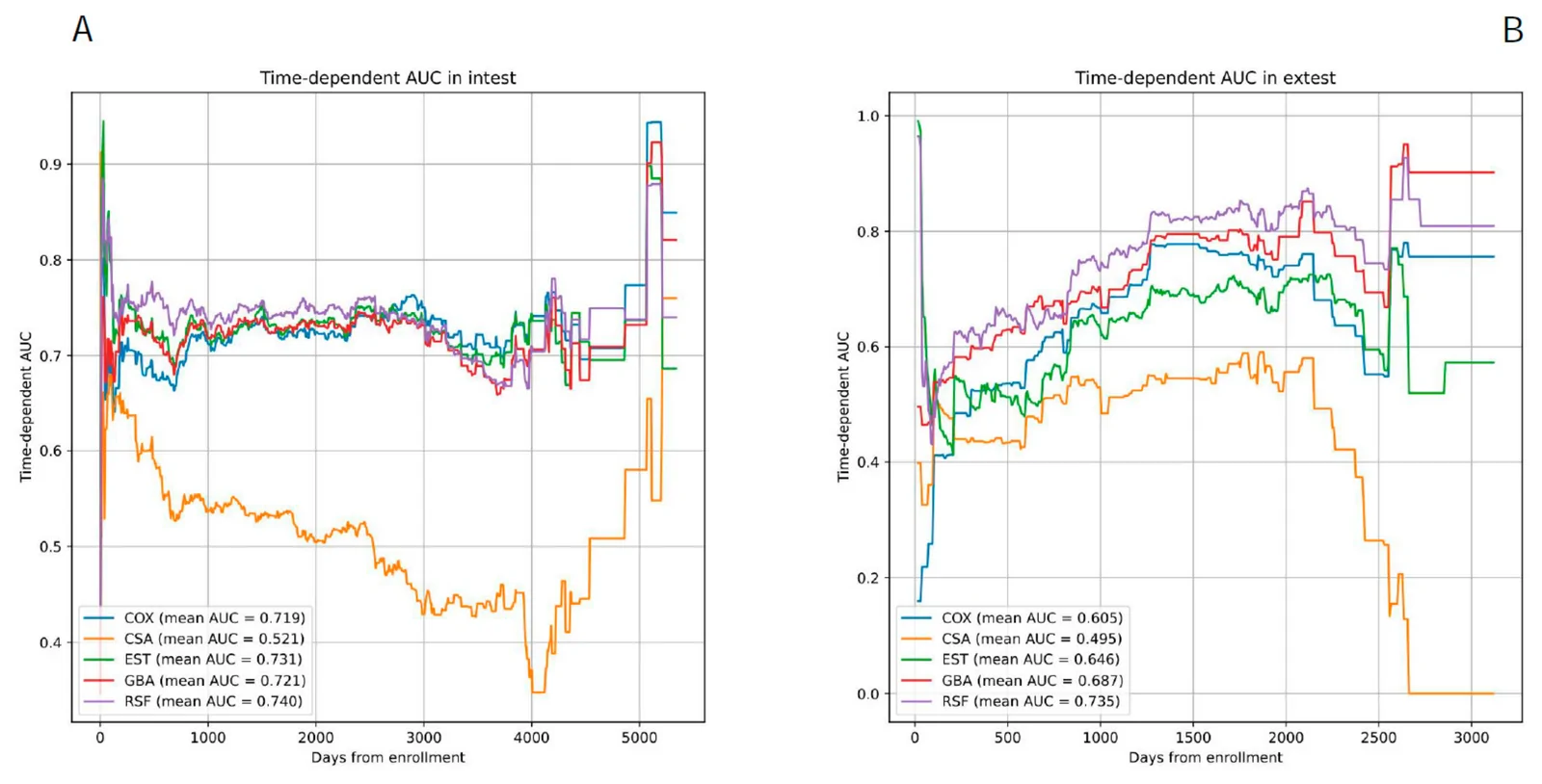
Time-dependent AUC curves for different models in internal and external validation. (**A**) Time-dependent AUC curves in the internal test cohort. (**B**) Time-dependent AUC curves in the external test cohort. COX, Cox Proportional Hazards Model; CSA, Coxnet Survival Analysis Model; EST, Extra Survival Trees; GBA, Gradient Boosting Survival Analysis; RSF, Random Survival Forest.

**Figure 3:**
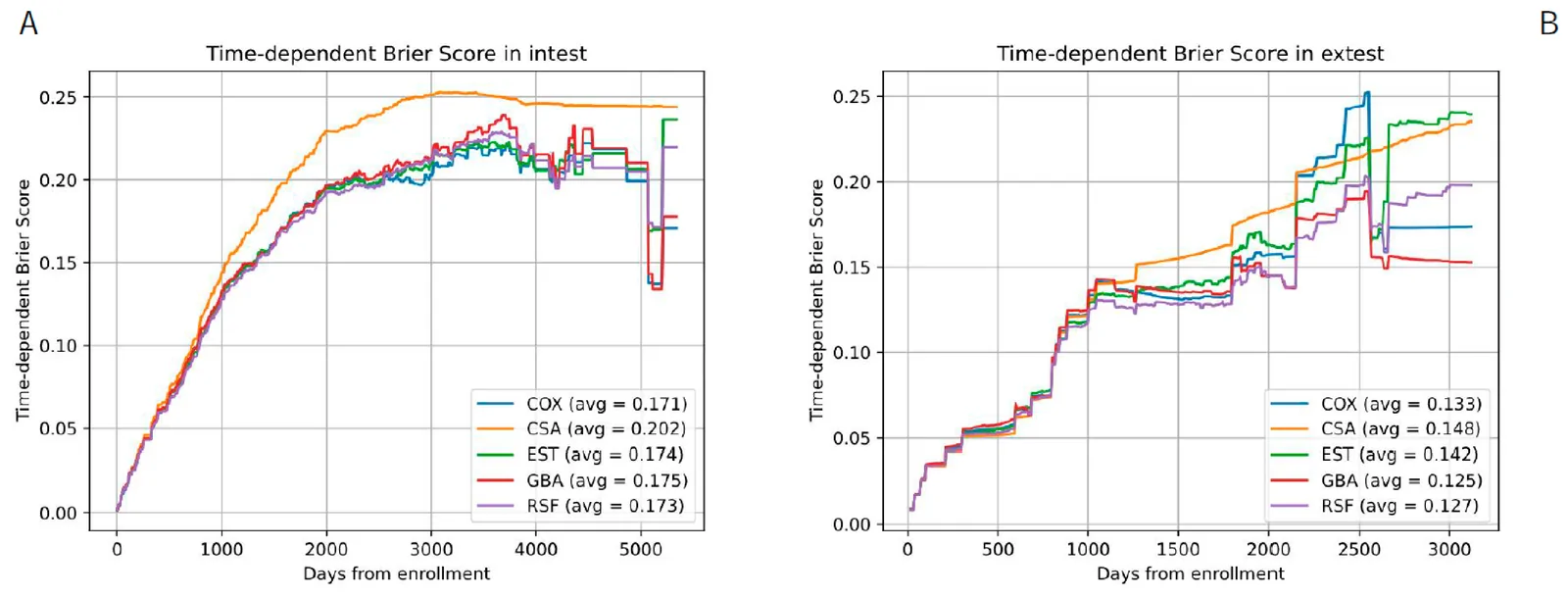
Time-dependent Brier scores for different models in internal and external validation. (**A**) Time-dependent Brier scores in the internal test cohort; (**B**) Time-dependent Brier scores in the external test cohort. COX, Cox Proportional Hazards Model; CSA, Coxnet Survival Analysis Model; EST, Extra Survival Trees; GBA, Gradient Boosting Survival Analysis; RSF, Random Survival Forest.

In the internal test set, the RSF model achieved the highest point estimates for discrimination, with a time-dependent AUROC of 0.74 (95% CI: 0.71, 0.77) and a C-index of 0.71 (95% CI: 0.67, 0.75), followed closely by EST, GBA, and COX (Figure 2A and Table 2). The 95% confidence intervals for these metrics substantially overlapped among RSF, COX, EST, and GBA, indicating that most differences may not be statistically significant except when compared with CSA. RSF also attained the lowest time-dependent Brier score (0.17, 95% CI: 0.16, 0.19) (Figure 3A and Table 2). Calibration analyses further demonstrated good agreement between predicted and observed mortality risks. Calibration plots closely followed the ideal reference line across 1-, 3-, and 5-year horizons (Figure S1A). Calibration slopes ranged from 1.117 to 1.356, and calibration intercepts ranged from 0.240 to 0.381 across the evaluated prediction horizons, while calibration-in-the-large values were close to zero. Observed-to-predicted mortality risk ratios were close to unity (0.962, 0.967, and 0.970 at 1-, 3-, and 5-year horizons, respectively) (Table S6). Furthermore, decile-based calibration analyses demonstrated a progressive increase in observed mortality risk across increasing predicted-risk groups, indicating preserved risk ordering across different levels of predicted mortality risk (Table S7).

In the external test cohort, RSF demonstrated favorable numerical performance compared with other models, achieving the highest time-dependent AUROC of 0.74 (95% CI:0.57, 0.86) (Figure 2B) and a C-index of 0.70 (95% CI: 0.58, 0.82) (Table 2), as well as a time-dependent Brier score of 0.13 (95% CI:0.10, 0.15) (Figure 3B). Calibration performance showed greater variability, particularly at longer prediction horizons, likely reflecting the limited sample size and follow-up duration. Only 26 patients remained at risk at 5 years (Table S4), resulting in less stable long-term calibration estimates. The 5-year calibration slope and calibration intercept were 2.256 and 0.854, respectively (Table S6). Nevertheless, calibration plots suggested that the overall relationship between predicted and observed risks was generally preserved, although greater deviations from the ideal reference line were observed compared with the internal test cohort (Figure S1B). Decile-based analyses in the external test cohort showed a similar trend between predicted and observed mortality risks, although estimates were less stable because of the limited sample size and number of events (Table S8).

**Figure 4:**
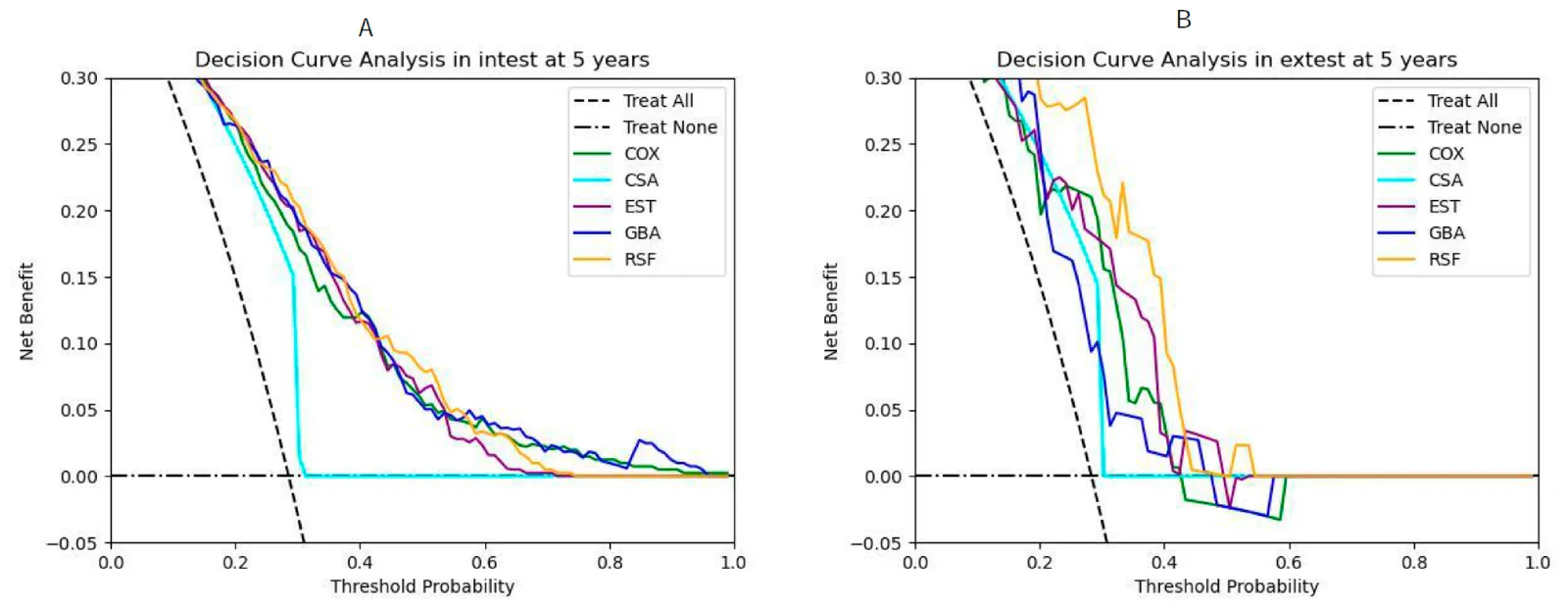
Decision curve analysis (DCA) for different models at 5 years in the internal and external test cohorts. (**A**) DCA curves in the internal test cohort; (**B**) DCA curves in the external test cohort. COX, Cox Proportional Hazards Model; CSA, Coxnet Survival Analysis Model; EST, Extra Survival Trees; GBA, Gradient Boosting Survival Analysis; RSF, Random Survival Forest.

In the 5-year DCA, the RSF model demonstrated numerically higher net benefit than other models across a range of threshold probabilities; however, these findings should be interpreted cautiously given the limited sample size of the external test cohort (Figure 4).

To further contextualize the performance of RSF, we conducted a sensitivity analysis comparing it with the established MADIT II score, which served as a clinical benchmark. As shown in Table 3, the RSF model achieved a higher C-index than MADIT II (0.70 vs. 0.58; 95% CI: 0.58, 0.82 and 0.44, 0.72; *p* = 0.039). The net reclassification index (NRI) at 5 years was 0.66 (95% CI: 0.47, 0.84) (Table 3), suggesting improved risk classification compared with the MADIT II score in this cohort.

Subgroup analyses showed that the RSF model performed robustly across most clinical subgroups, although discrimination was lower in the ischemic cardiomyopathy subgroup. Detailed results are presented in the Supplementary Materials (Table S9, Figures S2–S10).

To evaluate potential temporal variation in model performance, an era-based sensitivity analysis was performed according to the median implantation date (13 September 2016). RSF performance metrics, including C-index, time-dependent AUROC, and Brier score, were evaluated in the internal and external test cohorts (Table S10). Numerical variation in discrimination metrics was observed across implantation eras, whereas Brier scores did not show an increasing trend in later periods.

### 3.3. Survival Analysis

**Figure 5:**
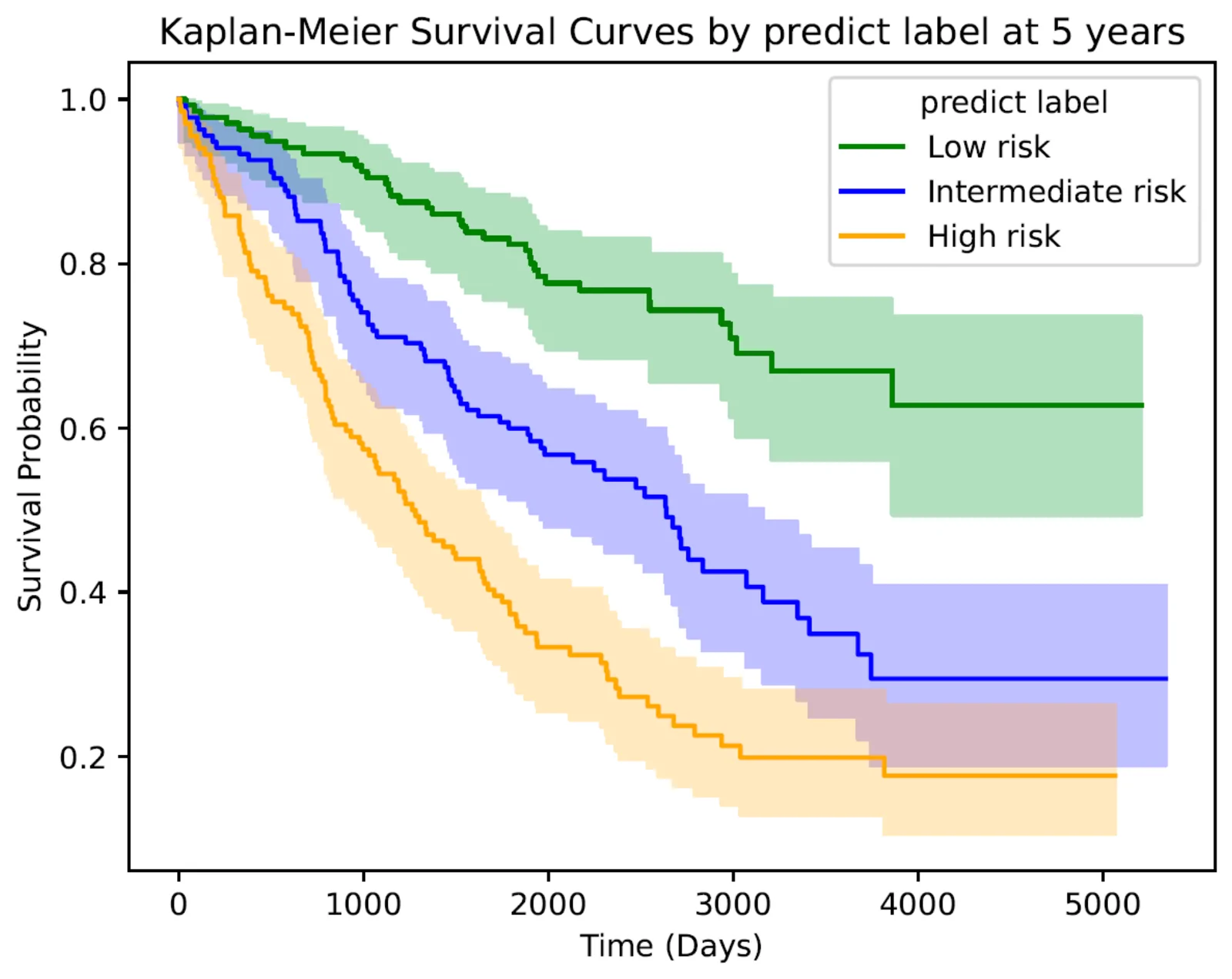
Kaplan–Meier survival curves for the low-, intermediate-, and high-risk subgroups, stratified by the RSF model’s predicted survival probabilities at the 5-year time point.

Patients were stratified into high-, intermediate-, and low-risk groups based on 5-year survival probabilities predicted by the RSF model. Kaplan–Meier curves demonstrated distinct survival trajectories, with high-risk patients showing a significantly higher mortality risk than low-risk patients (HR: 4.50, 95% CI: 3.10, 6.55, *p* < 0.0001), while intermediate-risk patients also had increased mortality (HR: 2.49, 95% CI: 1.69, 3.68, *p* < 0.0001) (Figure 5).

### 3.4. Interpretability Analysis

**Figure 6:**
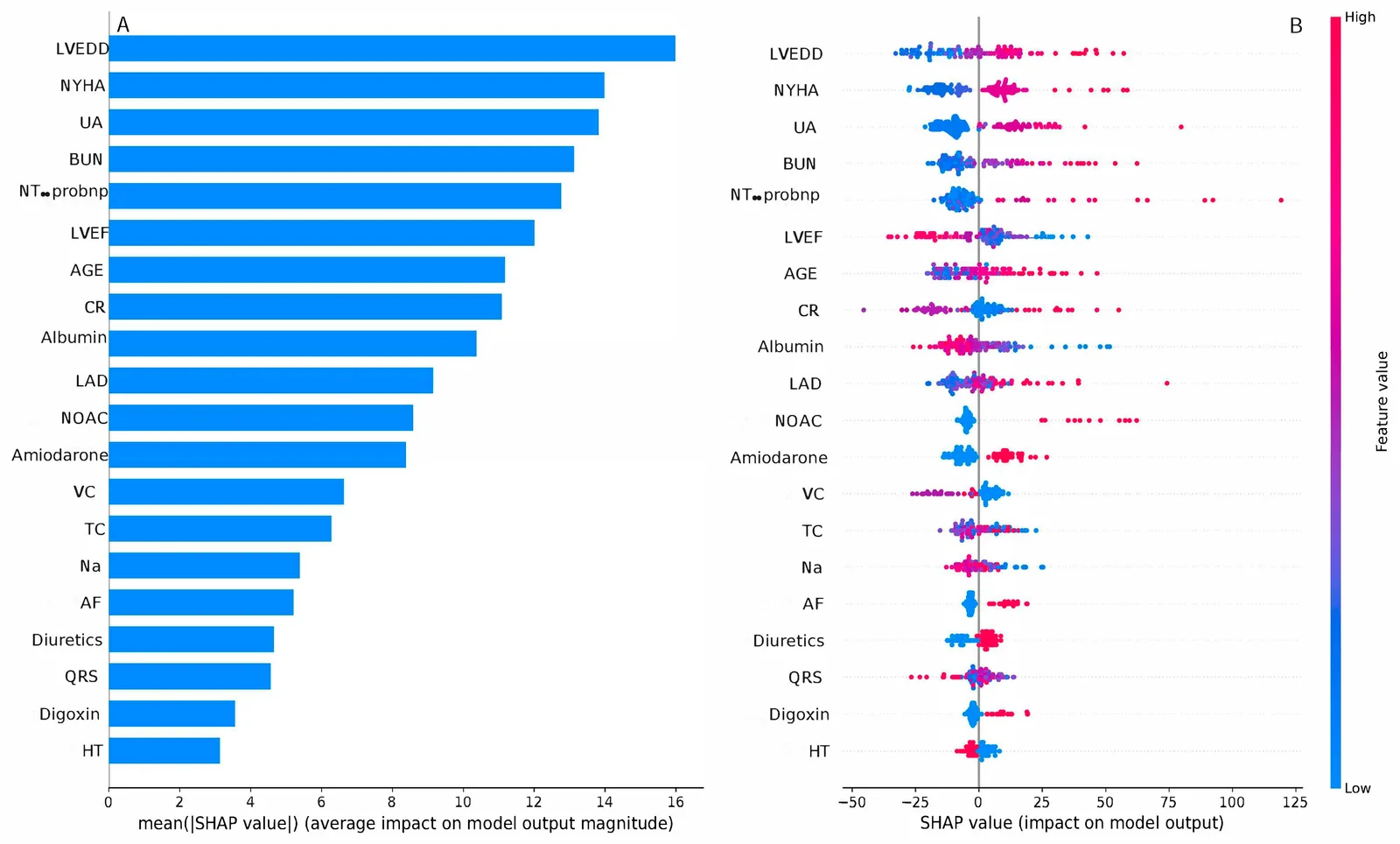
The model’s interpretation. (**A**) The ranking of the twenty most important features according to the mean (|SHAP value|); (**B**) The importance ranking of the twenty factors with stability and interpretation using the RSF model. Higher SHAP values indicate a greater contribution of that feature to the predicted mortality risk. The red and blue parts in feature value represent higher value and lower value. VC, Ventricular conduction; TC, Total Cholesterol; AF, Atrial fibrillation; QRS, QRS duration; HT, Hypertension.

Given that RSF demonstrated favorable numerical performance, Figure 6 further illustrates the significance of key features in determining patient outcomes. Notably, it summarizes the 20 most important predictors, with LVEDD emerging as the most influential factor. LVEDD demonstrated the highest average SHAP value, indicating its substantial impact on risk estimation.

### 3.5. Model Utility

We developed a web-based research prototype to facilitate demonstration and reproducible evaluation of the RSF model. Users can access the system via https://smirk-stranger-mug.ngrok-free.dev (accessed on 28 February 2025). By inputting predefined feature values, the system generates individualized survival probability estimates across a predefined 10-year prediction horizon following ICD/CRT-D implantation. However, the reliability of these estimates may vary over time, and longer-term predictions should be interpreted cautiously because fewer patients remain at risk at later follow-up points. The platform was implemented using a Flask-based framework. User-entered predictor variables are processed through the same preprocessing procedures used during model development and are then passed to the pretrained RSF model to generate survival probability estimates. The model parameters embedded in the tool are fixed and are not automatically updated. The deployed model represents a locked version, and future updates, if required, will be released as explicitly versioned models with corresponding validation procedures rather than automatic replacement of the existing model. The submitted feature values are processed only for prediction generation and are not stored. This prototype is intended for research and educational purposes.

## 4. Discussion

In this study, 5 ML survival models were developed to predict long-term mortality of patients receiving ICD or CRT-D implantation. In internal and external validation, the RSF model demonstrated favorable numerical performance across several evaluation metrics, including the time-dependent AUROC, C-index, and DCA, though confidence intervals overlapped with other models. The Brier score of RSF was identical to that of the standard Cox model, representing the lowest value among all models in the internal test set. The survival analysis further confirmed the model’s ability to effectively stratify patients into different risk groups. Additionally, interpretability analysis highlighted LVEDD as the most influential predictor, providing insights into the factors associated with mortality prediction and improving model interpretability. These findings may support future evaluation of the clinical relevance of the model pending further validation.

### 4.1. Advantages of the RSF Survival Model

The rapid growth of personalized and continuously collected data through digital health technologies, such as wearable devices and remote monitoring systems, has fueled interest in models capable of updating predictions over time [17,18,19]. While RSF does not incorporate real-time updates, it provides time-dependent survival estimates based on baseline data, offering flexible risk prediction across various time points. Unlike classification models that produce static, single-timepoint predictions and often oversimplify patient trajectories [12], RSF generates individualized survival probabilities over a continuous time horizon. This approach provides a more flexible understanding of patient risk trajectories and may facilitate future risk stratification strategies after further validation.

Traditionally, Cox proportional hazards (CPH) regression has been the standard for survival analysis but is limited by proportional hazard and linearity assumptions [20,21,22]. In contrast, RSF can potentially accommodate non-linear relationships and interactions among predictors. In this study, the RSF model achieved numerically higher mean AUCs than the Cox model in the internal test set (0.74 vs. 0.72) and external test set (0.74 vs. 0.61).

Several established risk models have been developed for mortality risk assessment in patients with heart failure or ICD implantation, including the Multicenter Automatic Defibrillator Implantation Trial II (MADIT II) score, the Seattle Heart Failure Model (SHFM), and the HF Meta-score [23,24,25]. In this study, MADIT II was selected as the primary reference model because it was developed in ICD recipients for mortality risk stratification, which more closely aligns with our objective of predicting long-term mortality after ICD/CRT-D implantation. In contrast, SHFM and HF Meta-score were primarily developed and validated in broader heart failure populations, with their main application focused on general heart failure prognosis and mortality risk assessment rather than individualized risk stratification after device implantation. Therefore, their target populations and intended clinical applications differ from the post-implantation risk assessment objective of the present study. Moreover, MADIT II shares several clinically relevant predictors with our model, supporting a meaningful comparison between conventional and machine learning-based risk models. However, MADIT II was derived from a more selected ICD population, and differences in patient characteristics should be considered when interpreting its performance in our heterogeneous ICD/CRT-D cohort.

Most previous studies have evaluated survival models using only one or two metrics, primarily the AUC or C-index, which may not fully capture model performance [8,20,21]. The present study incorporated all key evaluation metrics, including the C-index, AUROC, Brier score, and DCA, for a comprehensive assessment. The RSF model achieved the highest point estimates for most metrics, except for the Brier score where it ranked second in external tests. These findings suggest RSF provides favorable predictive performance, though further validation is needed.

### 4.2. Evaluation of Model Performance in Internal and External Tests

A significant limitation in current research is the lack of detailed reporting on machine learning methods, hindering reproducibility. Many studies also fail to adequately describe training and testing cohorts, reducing transparency [22]. Notably, Tse et al.’s study on SCD in Brugada syndrome is one of the few to include external validation, underscoring the need for more rigorous methodologies [23].

In this study, data from three of the four participating hospitals were used for model development and internal testing, while the fourth hospital served as an independent external test cohort to assess model performance in a separate clinical setting. This approach reduced the risk of data leakage and enabled an evaluation of the selected models using an independent cohort. Furthermore, systematic training, validation, and model optimization procedures were applied to establish a standardized modeling framework. The differences in baseline characteristics between the derivation and external validation cohorts may reflect real-world variations in patient characteristics, clinical profiles, and treatment patterns across clinical centers. However, these differences may influence model transportability and should be carefully considered when interpreting external validation performance across different populations. Furthermore, temporal changes in ICD/CRT-D management and heart failure therapies over the study period may represent additional sources of variation in patient risk profiles and model performance. Although era-based sensitivity analyses suggested potential temporal variation in discrimination performance across implantation eras, the impact of these temporal changes remains uncertain, particularly given the limited size of some era-specific subgroups and the external validation cohort. Further evaluation in contemporary and larger populations is warranted.

### 4.3. Risk Stratification Based on RSF Model Output

The Kaplan–Meier survival curves further highlight the RSF model’s ability to stratify patients into high-, intermediate-, and low-risk groups based on predicted survival probabilities. This stratification provides additional prognostic information and may facilitate individualized post-implantation risk assessment, allowing clinicians to identify patients who may require closer monitoring and more comprehensive clinical evaluation.

Importantly, the predicted mortality risk should be interpreted within the context of ICD/CRT-D therapy. Because the RSF model predicts all-cause mortality rather than arrhythmic mortality, a high predicted mortality risk does not necessarily indicate reduced potential benefit from ICD therapy and should not be used alone to determine whether ICD implantation should be withheld. Patients with high predicted mortality may have heterogeneous underlying risk profiles, including competing risks from non-arrhythmic causes of death. Therefore, decisions regarding ICD therapy should continue to incorporate comprehensive clinical assessment, including comorbidities, functional status, life expectancy, and guideline-based indications. Future studies incorporating cause-specific mortality and competing-risk analyses are warranted to further evaluate whether prognostic models can provide additional information for ICD decision-making.

### 4.4. Feature Selection and Interpretation

Interpretable ML techniques are essential for translating RSF predictions into clinical practice, as the lack of transparency in ML models (so-called ‘black box’ effect) remains a major concern. In the present study, LVEDD, NT-pro BNP, UA, BUN, and worsening heart function were identified by SHAP analysis as important contributors to model predictions of mortality risk. These findings are consistent with previous studies, which have demonstrated that impaired cardiac and renal function are strongly associated with increased mortality risk [24,25]. Notably, recent work by Katherine C. et al. reported that an elevated left ventricular end-diastolic volume (LVEDV) index serves as a highly reproducible and reliable prognostic marker, reinforcing our finding that LVEDD is the most significant predictor [6]. Some studies have suggested that left ventricular enlargement and remodeling are associated with adverse outcomes in heart failure [26,27,28,29,30]; however, the associations identified by SHAP analysis should not be interpreted as causal relationships or direct therapeutic targets.

Pharmacological interventions targeting LVEDV enlargement, including the “new quadruple therapy” (ARNI, beta-blockers, SGLT2 inhibitors, and spironolactone), have been shown to mitigate adverse cardiac remodeling [31]. Additionally, novel agents such as vericiguat have been introduced into clinical practice, offering new therapeutic options [32]. Early and comprehensive treatment strategies, particularly in high-risk patients, may delay HF progression, reduce the incidence of malignant arrhythmias, and ultimately improve long-term survival outcomes.

### 4.5. Future Perspectives of AI-Assisted Personalized ICD Management

Beyond post-implantation risk stratification, future personalized ICD management may benefit from dynamic assessment of arrhythmic risk across different stages of care. Wearable cardioverter-defibrillators combined with continuous monitoring and multiparametric reassessment have been shown to help optimize therapy and avoid unnecessary permanent ICD implantation in patients with transient or uncertain arrhythmic risk, highlighting the potential value of longitudinal risk evaluation rather than reliance solely on baseline characteristics [33].

Comprehensive ICD/CRT-D management should also consider device-related complications. Cardiac implantable electronic device infections are associated with substantial clinical and economic burdens, including prolonged hospitalization, antibiotic therapy, device extraction, morbidity, and mortality. Risk-based preventive strategies, including the use of gentamicin-impregnated collagen sponges in selected patients, may help reduce infection after device procedures, while systematic assessment of infection risk factors may facilitate targeted prevention strategies [34,35].

In parallel, artificial intelligence is increasingly being integrated into cardiac electrophysiology, with applications including ECG interpretation, arrhythmia detection, risk stratification, and clinical decision support [36]. Together, these developments suggest that AI-based approaches may complement existing clinical assessment by integrating multidimensional information to support personalized ICD management. However, prospective validation and careful evaluation of clinical implementation remain necessary before routine clinical adoption.

## 5. Limitations

This study has several limitations. First, due to its retrospective nature and data availability constraints, we were unable to incorporate all factors known to influence prognosis. Cause-specific mortality was not available in a standardized manner across participating centers; therefore, this study focused on all-cause mortality. Other clinically relevant outcomes, including heart transplantation, LVAD implantation, device-related events, appropriate ICD therapy, heart failure hospitalization, and sudden cardiac death, were not evaluated. In addition, the underlying mechanisms of missingness were not formally assessed, and different assumptions regarding missing data or imputation approaches may influence model performance. Second, the analysis was based on a single-modal dataset without incorporating multimodal data such as electrocardiographic signals or imaging features. Third, the baseline data were collected without dynamic follow-up measurements, limiting the model’s ability to capture temporal changes. Fourth, predictions beyond 6 years should be interpreted with caution due to the small number of patients remaining at risk. Subgroup analyses suggested that model performance varied across clinical populations, with weaker discrimination in the ischemic cardiomyopathy subgroup. Fifth, in addition, the web-based tool developed in this study represents a research prototype rather than a clinically validated decision-support system. Further prospective validation, usability testing, and assessment of privacy and cybersecurity requirements are warranted before potential clinical implementation. Finally, the external validation cohort was relatively small and demographically different from the development cohort; therefore, the external validation findings should be considered preliminary and interpreted cautiously. Future studies with larger, more diverse external populations are needed to fully assess the model’s clinical applicability.

## 6. Conclusions

Our study demonstrates the potential of an RSF survival model to provide individualized, time-resolved predictions of all-cause mortality risk after ICD and CRT-D implantation, supporting personalized risk stratification. Longer-term predictions should be interpreted cautiously due to the limited number of patients remaining at risk. Further prospective studies and randomized trials are warranted to validate its performance across diverse populations and clinical settings.

## Figures and Tables

**Table 1:** Baseline characteristics by derivation and external test cohorts.

Characteristic	All Patients (*n* = 3321)	Derivation Cohort (*n* = 3207)	External Test Cohort (*n* = 114)	*p* Value
**Demographic characteristics**				
Age, years	61.01 (51.01, 68.04)	61.02 (51.03, 68.01)	70.02 (63.01, 75.03)	<0.01
Male	2429 (73.14%)	2348 (73.21%)	81 (71.05%)	0.686
**Biochemical tests**				
Hemoglobin, g/L	138.01 (127.04, 149.03)	138.02 (127.03, 150.01)	134.31 (125.25, 145.75)	0.068
Albumin, g/L	41.01 (38.50, 43.15)	41.02 (38.51, 43.22)	40.09 (38.01, 43.03)	0.237
Bun, μmol/L	6.71 (5.34, 8.53)	6.65 (5.32, 8.51)	7.25 (5.91, 9.02)	0.758
Creatinine, μmol/L	86.01 (72.85, 103.02)	87.01 (73.35, 103.02)	30.39 (18.00, 75.01)	<0.01
Uric acid, μmol/L	393.02 (317.01, 464.02)	396.01 (323.03, 465.02)	370.02 (308.51,460.03)	0.541
Total cholesterol, mmol/L	4.02 (3.38, 4.59)	4.01(3.38, 4.58)	4.08 (3.53, 4.8175)	0.089
Na, mmol/L	140.25 (139.02, 142.01)	140.22 (139.03, 142.02)	141.01 (139.03, 144.01)	<0.01
NT-proBNP, pg/mL	1019.01 (387.02, 2434.01)	1052.01 (405.02, 2477.60)	381.53 (141.75, 964.52)	<0.01
**Echocardiographic parameters**				
LAD, mm	44.01 (38.02, 50.03)	44.02 (38.01, 50.03)	41.03 (38.02, 46.92)	<0.01
LVEDD, mm	60.23 ± 12.76	60.30 ± 12.80	58.37 ± 11.32	0.112
LVEF, %	37.02 (30.01, 55.04)	37.02 (30.03, 55.01)	38.02 (31.25, 57.51)	0.408
**Past medical history**				
Single/dual-chamber ICD	2209 (66.52%)	2143 (66.82%)	66 (57.89%)	0.060
Prior CIED implantation	59 (1.78%)	58 (1.81%)	1 (0.88%)	0.705
AVN ablation	88 (2.65%)	87 (2.71%)	1 (0.88%)	0.367
Primary prevention of SCD	2235 (67.30%)	2159 (67.32%)	76 (66.67%)	0.964
Ischemic etiology	763 (22.98%)	733 (22.86%)	30 (26.32%)	0.454
PCI	595 (17.92%)	577 (17.99%)	18 (15.79%)	0.632
CABG	85 (2.56%)	85 (2.65%)	0 (0.00%)	0.145
Hypertension	1307 (39.36%)	1253 (39.07%)	54 (47.37%)	0.092
Diabetes	661 (19.90%)	636 (19.83%)	25 (21.93%)	0.666
Atrial fibrillation	769 (23.16%)	743 (23.17%)	26 (22.81%)	1.0
Stroke	201 (6.05%)	194 (6.05%)	7 (6.14%)	1.0
Smoke	1127 (33.94%)	1093 (34.08%)	34 (29.82%)	0.399
Alcohol	778 (23.43%)	758 (23.64%)	20 (17.54%)	0.163
**Guide- directed medicine treatments, *n* (%)**				
Diuretics	2136 (64.32%)	2061 (64.27%)	75 (65.79%)	0.815
ACEI	1469 (44.23%)	1447 (45.12%)	22 (19.30%)	<0.01
ARNI	371 (11.17%)	330 (10.29%)	41 (35.96%)	<0.01
β-blockers	2706 (81.48%)	2645 (82.48%)	61 (53.51%)	<0.01
Aldosterone Receptor Antagonists	2133 (64.23%)	2064 (64.36%)	69 (60.53%)	0.460
Ivabradine	64 (1.93%)	64 (2.00%)	0 (0.00%)	0.239
Digoxin	833 (25.08%)	812 (25.32%)	21 (18.42%)	0.119
Amiodarone	1181 (35.56%)	1140 (35.55%)	41 (35.96%)	1.0
Statins	1275 (38.39%)	1216 (37.92%)	59 (51.75%)	<0.01
Warfarin	358 (10.78%)	353 (11.01%)	5 (4.39%)	0.037
NOAC	176 (5.30%)	165 (5.14%)	11 (9.65%)	0.058
**Clinical status**				
NYHA class III/IV	1401 (42.19%)	1344 (41.91%)	57 (50.00%)	0.292
**Electrocardiographic parameters**				
QRS duration, ms	120.01 (105.91, 157.02)	120.02 (105.91, 158.03)	128.90 (109.34, 146.65)	0.565
AV conduction anomaly	2824 (85.03%)	2718 (84.75%)	106 (92.98%)	0.064
Ventricular conduction anomaly	2019 (60.79%)	1949 (60.77%)	70 (61.40%)	0.535

Values are % or mean or median (IQR). ACEI, angiotensin-converting enzyme inhibitor; ARNI, Angiotensin Receptor-Neprilysin Inhibitor, atrioventricular node; AVN, Atrioventricular node; BUN, Blood Urea Nitrogen; CABG, coronary artery bypass graft; LAD, Left atrial diameter; LVEDD, Left ventricular end-diastolic diameter; LVEF, Left ventricular ejection fraction; NOAC, Non-Vitamin K Antagonist Oral Anticoagulant; NYHA, New York Heart Association; PCI, percutaneous coronary intervention; SCD: Sudden Cardiac Death.

**Table 2:** Performance of the models for predicting all-cause mortality.

Test Set	Metric	COX	CSA	EST	GBA	RSF
**Internal Test**	**C-index**	0.69 (0.65, 0.73)	0.52 (0.48, 0.57)	0.70 (0.66, 0.73)	0.70 (0.66, 0.74)	0.71 (0.67, 0.75)
	**Time-dependent AUROC**	0.72 (0.69, 0.75)	0.52 (0.46, 0.54)	0.73 (0.72, 0.77)	0.72 (0.68, 0.73)	0.74 (0.71, 0.77)
	**Time-dependent Brier**	0.17 (0.16, 0.19)	0.20 (0.20, 0.21)	0.17 (0.17, 0.19)	0.18 (0.15, 0.20)	0.17 (0.16, 0.19)
**External Test**	**C-index**	0.62 (0.47, 0.78)	0.51 (0.39, 0.64)	0.61 (0.47, 0.76)	0.63 (0.53, 0.74)	0.70 (0.58, 0.82)
	**Time-dependent AUROC**	0.61 (0.46, 0.72)	0.49 (0.39, 0.56)	0.65 (0.47, 0.74)	0.69 (0.57, 0.73)	0.74 (0.57, 0.86)
	**Time-dependent Brier**	0.13 (0.11, 0.17)	0.15 (0.12, 0.16)	0.14 (0.12, 0.15)	0.12 (0.09, 0.16)	0.13 (0.09, 0.15)

Values are estimates with 95% confidence intervals. COX, Cox Proportional Hazards Model; CSA, Coxnet Survival Analysis Model; EST, Extra Survival Trees; GBA, Gradient Boosting Survival Analysis; RSF, Random Survival Forest; AUROC, area under the receiver operating characteristic curve.

**Table 3:** Sensitivity analysis—Comparison with MADIT II score.

Outcome	Baseline	RSF	*p* Value	NRI
Mortality	0.58 (0.44, 0.72)	0.70 (0.58, 0.82)	0.039	0.66 (0.47, 0.84)

Cox models were fit using the variables listed, and the C-index was evaluated for baseline model (MADIT II score) and RSF model. In addition, the net reclassification index (NRI) was calculated to quantify the improvement in risk stratification with RSF compared with baseline model. Baseline model: MADIT II score: NYHA class, age, blood urea nitrogen, QRS duration, atrial fibrillation/flutter.

## Data Availability

The data that support the findings of this study are not publicly available due to privacy or ethical restrictions but are available from the corresponding author upon reasonable request. The source code used for model development, validation, prediction, and interpretation analyses is available at GitHub: https://github.com/zs-xinsight/pacemaker-death (accessed on 16 August 2026).

## Supplementary Materials

The following supporting information can be downloaded at https://www.mdpi.com/article/10.3390/jcdd13100514/s1, Supplementary S1: Data collection details; Table S1: Missingness of candidate predictors before imputation; Table S2: List of clinical predictors selected for survival model development; Table S3: The best-tuned hyperparameters for each model; Table S4: Survival characteristics across training, internal test, and external test cohorts; Table S5: Device characteristics and clinical indications across training, internal test, and external test cohorts; Table S6: Calibration performance of the RSF model at different prediction horizons; Table S7: Observed versus predicted mortality risk across deciles of predicted risk from the RSF model in the internal test cohort; Table S8: Observed versus predicted mortality risk across deciles of predicted risk from the RSF model in the external test cohort; Table S9: Performance of the models for predicting all-cause mortality in different subgroups; Table S10: Era-based sensitivity analysis of RSF performance according to implantation era; Figure S1: Calibration plots of the RSF model at different prediction horizons; Figure S2: Time-dependent AUC for the RSF model in primary and secondary ICD; Figure S3: Kaplan–Meier survival curves stratified by the RSF model’s predicted survival probabilities at the 5-year time point in primary and secondary ICD; Figure S4: Time-dependent AUC curves for different models in primary and secondary ICD; Figure S5: Time-dependent AUC in ICD and CRT-D; Figure S6: Kaplan–Meier survival curves stratified by the RSF model’s predicted survival probabilities at the 5-year time point in ICD and CRT-D; Figure S7: Time-dependent AUC curves for different models in ICD and CRT-D; Figure S8: Time-dependent AUC in ischemic and nonischemic cardiomyopathy; Figure S9: Kaplan–Meier survival curves stratified by the RSF model’s predicted survival probabilities at the 5-year time point in ischemic and nonischemic cardiomyopathy; Figure S10: Time-dependent AUC curves for different models in ischemic and nonischemic cardiomyopathy.
