# Supplementary material for: Machine Learning-Based Prediction of Long-Term Mortality in Patients with Implantable Cardioverter Defibrillators
Source: J Cardiovasc Dev Dis. 2026 Oct 12;13(10):514. doi: 10.3390/jcdd13100514 (PMC13705327; doi:10.3390/jcdd13100514)

Supplementary materials

for 'Development and evaluation of prognostic models for mortality of patients receiving implantable cardioverter defibrillators with machine learning' by Lei Pan, et al.

Supplementary S1. Data collection details

Data at the time of implantation procedure retrospectively collected includes:

1) Demographic characteristics, including age and sex.

2) Biochemical tests, including hemoglobin, Lymphocytes, albumin, blood urea nitrogen, serum creatinine, serum uric acid, total cholesterol, sodium, N-terminal prohormone of brain natriuretic peptide, Estimated glomerular filtration rate (eGFR) was calculated by the elaborate MDRD formula based on the value of serum albumin, serum creatinine, serum uric acid, and adjusted by the patient's age and sex.

3) Echocardiographic parameters, including left atrial diameter (LAD), left ventricular end-diastolic diameter (LVEDD), left ventricular ejection fraction (LVEF).

4) Past medical history, including Single or dual-chamber ICD, prior cardiac implantable electronic device implantation, Atrioventricular node ablation, history of sudden cardiac death, etiology (ischemic or non-ischemic), history of percutaneous coronary intervention , history of coronary artery bypass grafting, hypertension, diabetes mellitus, atrial fibrillation, Stroke, Smoke, Alcohol .Etiology was considered ischemic in the presence of significant coronary artery disease (≥50% stenosis in 1 or more of the major coronary arteries), history of myocardial infarction, or prior revascularization.

5) Medication at discharge after index implantation procedure, including diuretics, angiotensin converting

enzyme inhibitor (ACEI)/angiotensin II receptor blocker (ARB)/angiotensin receptor-neprilysin inhibitor (ARNI), β-blocker, Aldosterone Receptor Antagonists, ivabradine, digoxin, amiodarone, statins, warfarin, and Non-Vitamin K Antagonist Oral Anticoagulant.

6) Electrocardiographic parameters, including atrioventricular conduction anomaly, ventricular conduction anomaly, QRS duration. Atrioventricular conduction anomaly include First-degree atrioventricular , Second-degree atrioventricular block, Third-degree atrioventricular block. Ventricular conduction anomaly was categorized as left bundle branch block (LBBB), right bundle branch block (RBBB) and non-specific interventricular conduction delay (IVCD).

7) Clinical status, including New York Heart Association functional class.

Table S1. Missingness of candidate predictors before imputation

Candidate predictor	Training cohort, n(%)	Internal test cohort, n (%)	External test cohort, n (%)
ACEI	0 (0.0%)	0 (0.0%)	0 (0.0%)
Atrial fibrillation	2 (0.08%)	0 (0.0%)	0 (0.0%)
Age	7 (0.27%)	2 (0.31%)	0 (0.0%)
Albumin	317 (12.35%)	91 (14.2%)	4 (3.51%)
Alcohol	1 (0.04%)	0 (0.0%)	0 (0.0%)
Aldosterone Receptor Antagonists	1 (0.04%)	0 (0.0%)	0 (0.0%)
Amiodarone	34 (1.33%)	9 (1.4%)	0 (0.0%)
ARNI	0 (0.0%)	0 (0.0%)	0 (0.0%)
AVconduction anomaly	49 (1.91%)	12 (1.87%)	54 (47.37%)
AVN ablation	0 (0.0%)	0 (0.0%)	0 (0.0%)
β-blockers	0 (0.0%)	0 (0.0%)	0 (0.0%)
Ventricular conduction anomaly	44 (1.71%)	11 (1.72%)	54 (47.37%)
Bun	2 (0.08%)	0 (0.0%)	1 (0.88%)
CABG	0 (0.0%)	0 (0.0%)	0 (0.0%)
Creatinine	2 (0.08%)	0 (0.0%)	1 (0.88%)
Single/dual-chamber ICD	35 (1.36%)	12 (1.87%)	0 (0.0%)
Digoxin	0 (0.0%)	0 (0.0%)	0 (0.0%)
Diuretics	0 (0.0%)	0 (0.0%)	0 (0.0%)

Diabetes	0 (0.0%)	0 (0.0%)	0 (0.0%)
Etiology	0 (0.0%)	0 (0.0%)	0 (0.0%)
Hemoglobin	3 (0.12%)	3 (0.47%)	1 (0.88%)
Hypertension	0 (0.0%)	0 (0.0%)	0 (0.0%)
Ivabradine	13 (0.51%)	6 (0.94%)	0 (0.0%)
LAD	60 (2.34%)	10 (1.56%)	10 (8.77%)
LVEDD	50 (1.95%)	9 (1.4%)	8 (7.02%)
LVEF	27 (1.05%)	4 (0.62%)	8 (7.02%)
Na	323 (12.59%)	92 (14.35%)	2 (1.75%)
NOAC	0 (0.0%)	0 (0.0%)	0 (0.0%)
NYHA	2 (0.08%)	1 (0.16%)	0 (0.0%)
PCI	0 (0.0%)	0 (0.0%)	0 (0.0%)
Prevention of SCD	198 (7.72%)	45 (7.02%)	0 (0.0%)
PriorCIED implantation	0 (0.0%)	0 (0.0%)	0 (0.0%)
NT-proBNP	80 (3.12%)	21 (3.28%)	8 (7.02%)
QRS duration	464 (18.08%)	117 (18.25%)	54 (47.37%)
Sex	3 (0.12%)	0 (0.0%)	0 (0.0%)
Smoke	1 (0.04%)	0 (0.0%)	0 (0.0%)
Statins	37 (1.44%)	15 (2.34%)	0 (0.0%)
Stroke	0 (0.0%)	0 (0.0%)	0 (0.0%)
Total cholesterol	320 (12.47%)	80 (12.48%)	8 (7.02%)
Uric acid	187 (7.29%)	55 (8.58%)	1 (0.88%)
Warfarin	0 (0.0%)	0 (0.0%)	0 (0.0%)

Missing values are presented as number of patients with missing data (percentage). Variables with missingness >60% in the training cohort were excluded before imputation.

ACEI, angiotensin-converting enzyme inhibitor; ARNI, Angiotensin Receptor-Neprilysin Inhibitor, atrioventricular node; AVN, Atrioventricular node; Bun, Blood Urea Nitrogen; CABG, coronary artery bypass graft; LAD, Left atrial diameter; LVEDD, Left ventricular end-diastolic diameter; LVEF, Left ventricular ejection fraction; NOAC, Non-Vitamin K Antagonist Oral Anticoagulant; NYHA, New York Heart Association; PCI, percutaneous coronary intervention;

Table S2 List of clinical predictors selected for survival model development

Variable category	Final selected clinical predictors
Demographic characteristics	Age; Sex
Laboratory measurements	Albumin; Blood urea nitrogen; Creatinine; Uric acid; Total cholesterol; Sodium; NT-proBNP
Electrocardiographic parameters	QRS duration; AV conduction anomaly; Ventricular conduction anomaly
Echocardiographic parameters	Left atrial diameter; Left ventricular end-diastolic diameter; Left ventricular ejection fraction
Clinical history	Previous PCI; Hypertension; Diabetes mellitus; Atrial fibrillation; Stroke;Prevention indication of SCD（primary vs secondary）

Medication use	Diuretics; Aldosterone receptor antagonists; Digoxin; Amiodarone; Warfarin; Non-vitamin K antagonist oral anticoagulants
Clinical status	NYHA class

SCD: Sudden Cardiac Death

Table S3 The best-tuned hyperparameters for each model

Survival models	Hyperparameters	The best-tuned hyperparameters
Cox Proportional Hazards Model	alpha:[0, 0.1, 0.01],	alpha: 0.1,
	n_iter:[200, 1000, 2000],	n_iter: 200,
	tol:[1e-5, 1e-6, 1e-7]	tol: 1e-6
Coxnet Survival Analysis	tol: [1e-6, 1e-4, 1e-10],	tol: 1e-10,
	l1_ratio: [0.5, 1, 0.1],	l1_ratio: 0.5,
	max_iter: [1000, 2000],	max_iter: 1000,
Extra Survival Trees	max_depth: [5, 50, 10, 20],	max_depth: 50,
	min_samples_leaf: [1, 2, 10, 5],	min_samples_leaf: 1,
	min_samples_split: [20, 10, 50]	min_samples_split: 10
Gradient Boosting Survival Analysis	n_estimators: [200, 100, 50],	n_estimators: 200,
	max_depth: [2, 3, 5],	max_depth: 3,
	min_samples_split: [2, 5, 1],	min_samples_split: 5,
	learning_rate: [0.1, 0.01, 0.001]	learning_rate: 0.1
Random Survival Forest	n_estimators': [100, 200],	n_estimators: 100,
	max_depth: [40, 20, 10, 5],	max_depth: 40,
	min_samples_split: [2, 5, 1, 10]	min_samples_split: 5,

Table S4. Survival characteristics across training, internal test, and external test cohorts

Variable	Training cohort	Internal test cohort	External test cohort
Patients, n	2566	641	114
All-cause deaths, n (%)	848 (33.0%)	216 (33.7%)	18 (15.8%)
Median follow-up, years (IQR)	4.10 (2.34–6.54)	3.99 (2.23–6.31)	3.15 (1.56–4.90)
Total person-years of follow-up	12158.2	2908	371.9
Number at risk at 1 year	2367	589	98
Number at risk at 3 years	1673	397	59
Number at risk at 5 years	1011	242	26
Number at risk at 10 years	196	44	0

Values are presented as number (%), median (interquartile range [IQR]), or total person-years, as appropriate. Number at risk represents the number of patients who remained under observation and were eligible for survival estimation at each specified time point.

Table S5. Device characteristics and clinical indications across training, internal test, and external test cohorts

Variable	Training cohort	Internal test cohort	External test cohort
ICD	1716 (66.9%)	428 (66.8%)	66 (57.9%)
CRT-D	850 (33.1%)	213 (33.2%)	48 (42.1%)
Primary prevention	1717 (66.9%)	446 (69.6%)	76 (66.7%)
Secondary prevention	849 (33.1%)	195 (30.4%)	38 (33.3%)
Ischemic etiology	605 (23.6%)	128 (20.0%)	30 (26.3%)
Nonischemic etiology	1961 (76.4%)	513 (80.0%)	84 (73.7%)

Values are presented as number (%). ICD indicates implantable cardioverter-defibrillator; CRT-D indicates cardiac resynchronization therapy with defibrillator.

Table S6. Calibration performance of the RSF model at different prediction horizons

Validation cohort	Prediction horizon	Observed-to-predicted mortality risk ratio	Calibration slope	Calibration intercept	Calibration-in-the-large
Internal test	1 years	0.962	1.117	0.24	-0.033
Internal test	3 years	0.967	1.284	0.381	0.013
Internal test	5 years	0.97	1.356	0.294	0.014
External test	1 years	1.034	0.364	-1.732	0.014
External test	3 years	0.899	1.342	0.416	-0.065

External test	5 years	0.692		2.256	0.854	-0.262

Table S7 Observed versus predicted mortality risk across deciles of predicted risk from the RSF model in the internal test cohort

Validation cohort	Prediction horizon (years)	Risk decile	Number of patients	Mean predicted mortality risk (%)	Observed mortality risk (%)
Internal test	1	D01	65	0.52	0
Internal test	1	D02	64	1.49	3.18
Internal test	1	D03	64	2.4	1.56
Internal test	1	D04	64	3.22	3.17
Internal test	1	D05	64	4.27	4.69
Internal test	1	D06	64	5.4	3.12
Internal test	1	D07	64	6.76	7.81
Internal test	1	D08	64	8.77	6.33
Internal test	1	D09	64	11.44	11.22
Internal test	1	D10	64	19.53	20.46
Internal test	3	D01	65	4.33	3.25
Internal test	3	D02	64	7.95	8.65
Internal test	3	D03	64	10.75	6.92
Internal test	3	D04	64	13.24	9.13
Internal test	3	D05	64	16	21.19
Internal test	3	D06	64	19.54	14.64
Internal test	3	D07	64	23.28	21.49
Internal test	3	D08	64	27.23	31.47
Internal test	3	D09	64	32.66	29.18
Internal test	3	D10	64	44.6	49.34
Internal test	5	D01	65	8.44	5.88
Internal test	5	D02	64	15.39	14.85
Internal test	5	D03	64	20.42	19.21
Internal test	5	D04	64	25.05	14.91
Internal test	5	D05	64	29.43	29.72
Internal test	5	D06	64	32.95	33.06
Internal test	5	D07	64	37.26	40.59
Internal test	5	D08	64	43.05	37.37
Internal test	5	D09	64	49.39	50.01
Internal test	5	D10	64	60.81	75.22

Values represent the mean predicted mortality risk and corresponding observed mortality risk estimated by the Kaplan–Meier method within each decile of RSF-predicted risk. Predicted risks were calculated at 1-, 3-, and 5-year horizons. Deciles were defined according to the distribution of predicted mortality risk in the internal test cohort**.**

Table S8 Observed versus predicted mortality risk across deciles of predicted risk from the RSF model in the external test cohort

Validation cohort	Prediction horizon (years)	Risk decile	Number of patients	Mean predicted mortality risk (%)	Observed mortality risk (%)
External test	1	D01	12	0.33	0
External test	1	D02	11	1.15	9.09
External test	1	D03	11	2.02	0
External test	1	D04	12	2.88	8.33
External test	1	D05	11	3.56	0
External test	1	D06	11	4.57	0
External test	1	D07	12	5.82	0
External test	1	D08	11	7.03	19.19
External test	1	D09	11	8.62	19.19
External test	1	D10	12	15.91	0
External test	3	D01	12	6.15	0
External test	3	D02	11	9.07	0
External test	3	D03	11	11.07	9.09
External test	3	D04	12	13.46	0
External test	3	D05	11	16.2	0
External test	3	D06	11	18.51	34.55
External test	3	D07	12	20.7	31.25
External test	3	D08	11	23.96	34.38
External test	3	D09	11	26.89	42.28
External test	3	D10	12	41.09	20
External test	5	D01	12	9.54	0
External test	5	D02	11	16.08	0
External test	5	D03	11	21.53	9.09
External test	5	D04	12	25.48	0
External test	5	D05	11	27.9	24.24
External test	5	D06	11	31.71	39.39
External test	5	D07	12	35.17	31.25
External test	5	D08	11	40.04	44.44
External test	5	D09	11	42.84	66.33
External test	5	D10	12	56.93	20

Values represent the mean predicted mortality risk and corresponding observed mortality risk estimated by the Kaplan–Meier method within each decile of RSF-predicted risk. Predicted risks were calculated at 1-, 3-, and 5-year horizons. Deciles were defined according to the distribution of predicted mortality risk in the external test cohort

Table S9. Performance of the models for predicting all-cause mortality in different subgroups: Primary and Secondary ICD,ICD and CRT-D,Ischemic CM and Nonischemic CM

	RSF	COX	CSA	EST	GBA
**Primary-ICD**					
C-index	0.73(0.69, 0.77)	0.70(0.66, 0.74)	0.50(0.45, 0.56)	0.71(0.66, 0.75)	0.71(0.66, 0.75)
P	/	0.012	<0.001	0.014	0.012
**Secondary-ICD**					
C-index	0.65(0.59, 0.72)	0.66(0.59, 0.73)	0.56(0.50, 0.63)	0.64(0.58, 0.71)	0.67(0.60, 0.73)
P	/	0.332	0.025	0.315	0.251
**ICD**					
C-index	0.69(0.65, 0.73)	0.67(0.63, 0.72)	0.56(0.51, 0.61)	0.66(0.62, 0.70)	0.68(0.64, 0.72)
P	/	0.108	<0.001	0.008	0.181
**CRT-D**					
C-Index	0.71(0.65, 0.78)	0.71(0.64, 0.78)	0.44(0.37, 0.52)	0.74(0.67, 0.80)	0.69(0.63, 0.76)
P	/	0.494	<0.001	0.151	0.242
**Ischemic CM**					
C-index	0.53(0.42, 0.63)	0.52(0.41, 0.63)	0.57(0.47, 0.67)	0.51(0.40, 0.61)	0.54(0.44, 0.65)
P	/	0.392	0.261	0.223	0.29
**Nonischemic CM**					
C-Index	0.74(0.70, 0.78)	0.72(0.68, 0.75)	0.51(0.47, 0.56)	0.72(0.69, 0.76)	0.72(0.68, 0.76)
P	/	0.028	<0.001	0.056	0.02

The table presents C-index values (95% confidence intervals) for five models in different subgroups. CM:Cardiomyopathy

Table S10. Era-based sensitivity analysis of RSF performance according to implantation era

Cohort	Implantation era	n	Events	C-index (95% CI)	Time-dependent AUROC (95% CI)	Brier score (95% CI)
Internal test cohort	Before 2016-09-13	321	136	0.720 (0.677–0.762)	0.772 (0.721–0.819)	0.173 (0.151–0.192)
Internal test cohort	2016-09-13 and after	320	80	0.601(0.535–0.654)	0.668 (0.583–0.729)	0.164 (0.134–0.192)
External test cohort	Before 2016-09-13	14	4	0.609 (0.179–1.000)	0.818 (0.154–1.000)	0.147 (0.100–0.249)
External test cohort	2016-09-13 and after	100	14	0.565 (0.424–0.718)	0.528 (0.351–0.704)	0.109 (0.070–0.150)

Implantation era was defined using the median implantation date (September 13, 2016) as the cutoff. Confidence intervals were calculated by bootstrap resampling.

Figure S1 Figure S1. Calibration plots of the RSF model at different prediction horizons. (A) Calibration plots at 1, 3, and 5 years in the internal test cohort; (B) Calibration plots at 1, 3, and 5 years in the external test cohort.

A

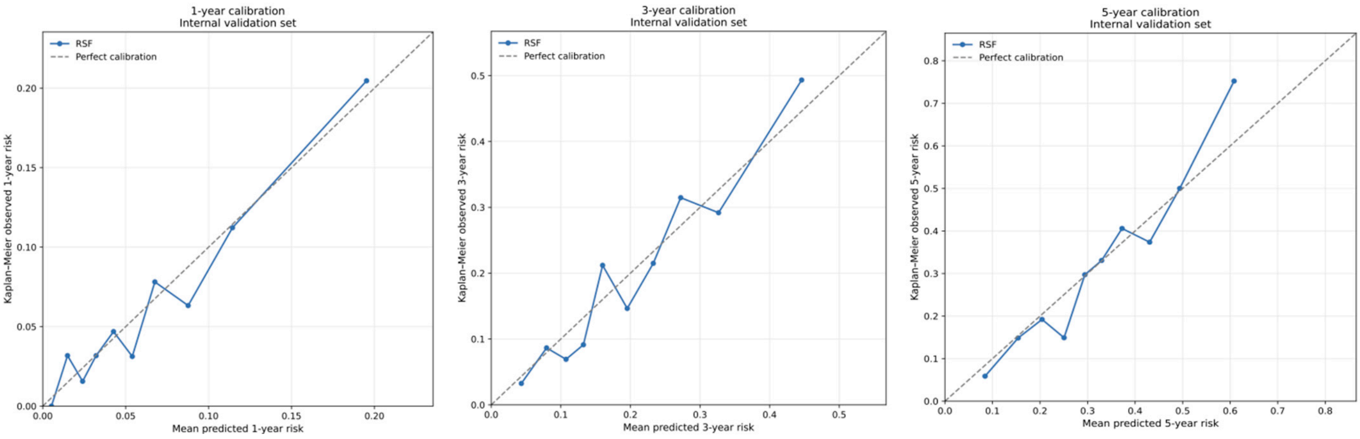

B

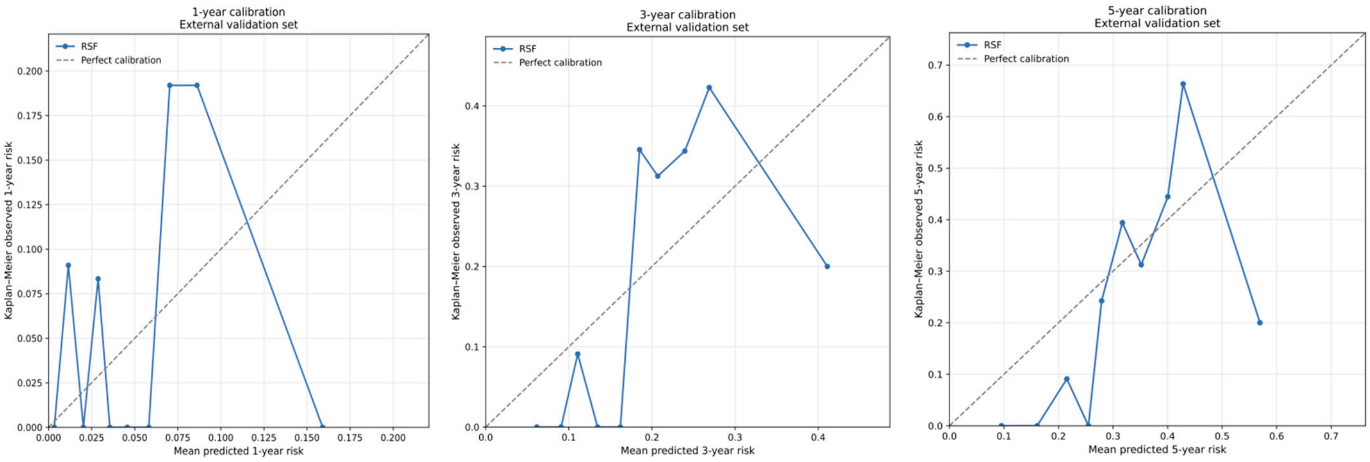

Figure S2. Time-dependent AUC for RSF model in Primary and Secondary ICD

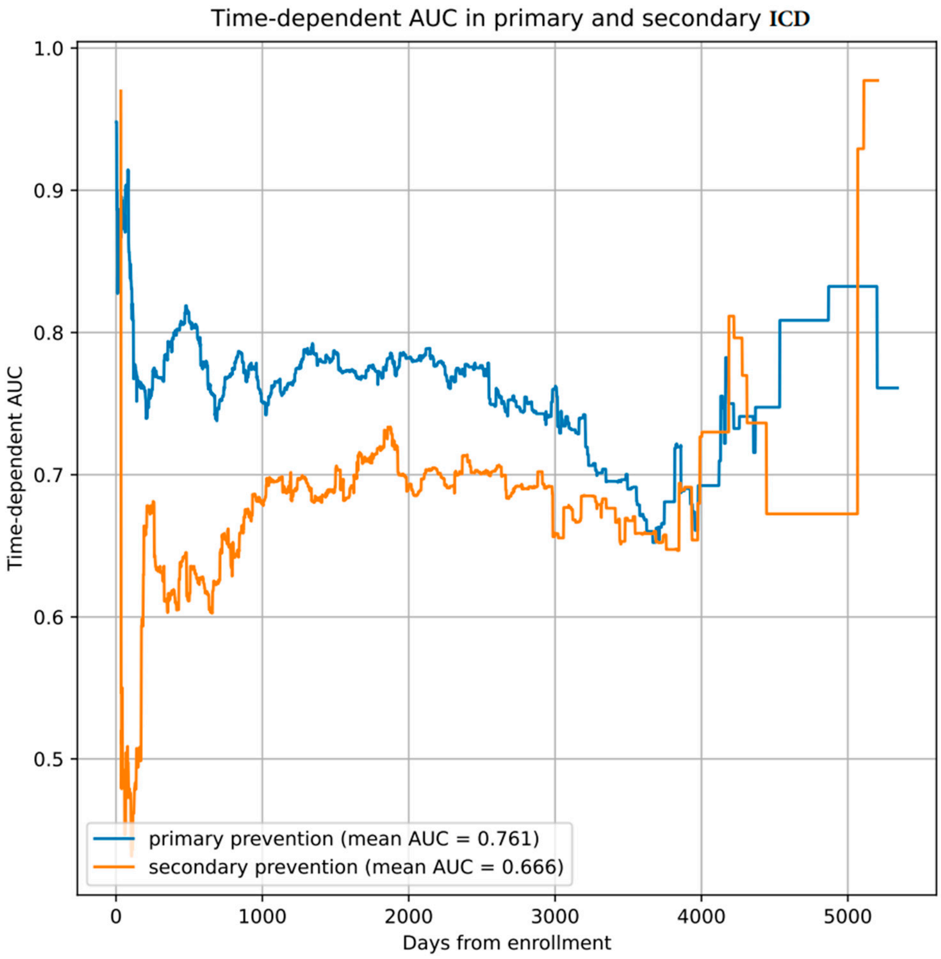

Figure S3. Kaplan – Meier survival curves stratified by the RSF model's predicted survival probabilities at the 5-year time point. (A) Primary ICD(B) Secondary ICD

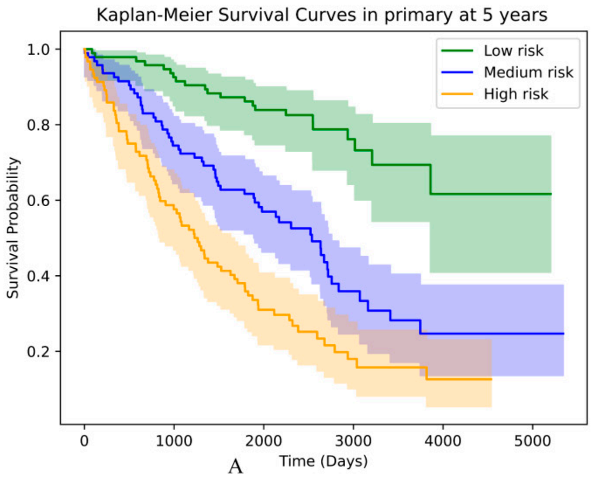

A

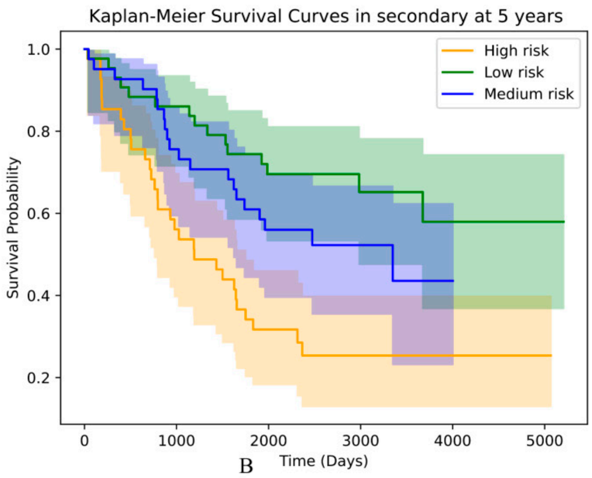

B

Figure S4. Time-dependent AUC curves for different models in (A) Primary ICD(B) Secondary ICD

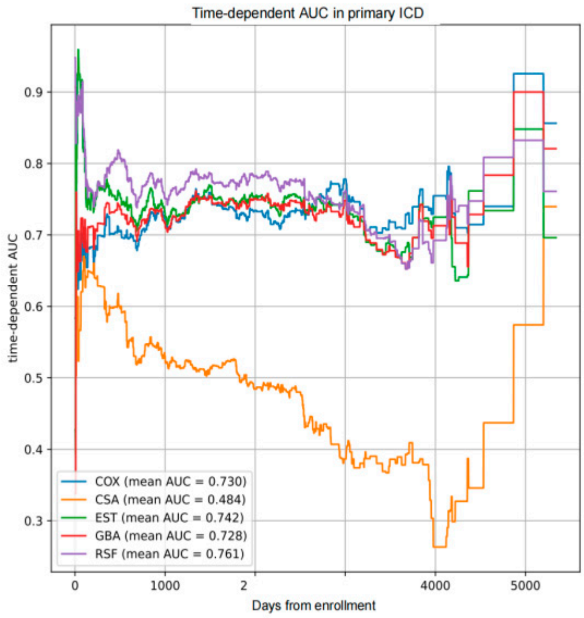

A

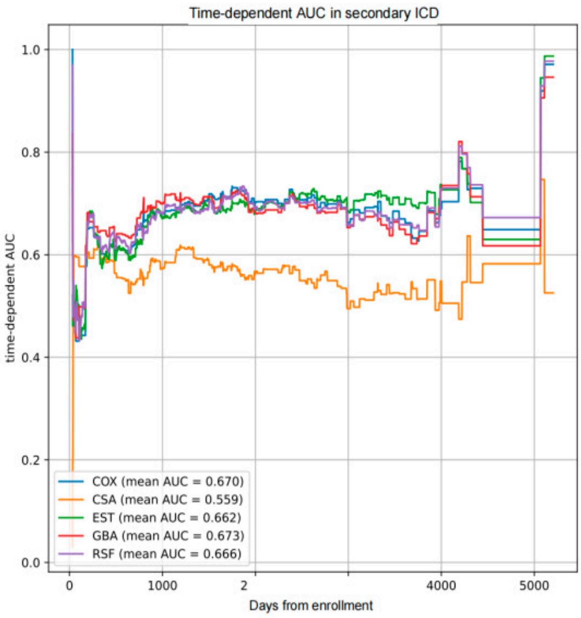

B

Figure S5. Time-dependent AUC in ICD and CRT-D

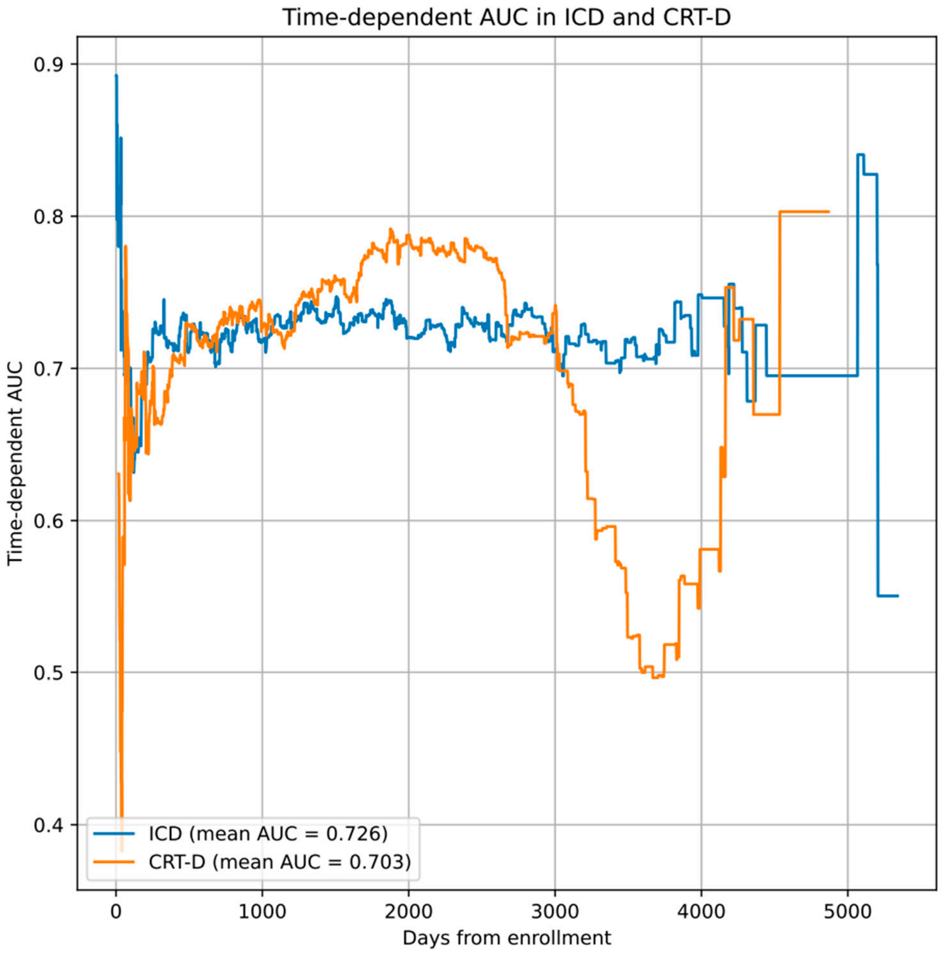

Figure S6. Kaplan－Meier survival curves stratified by the RSF model’ s predicted survival probabilities at the 5-year time point. (A) ICD (B) CRT-D

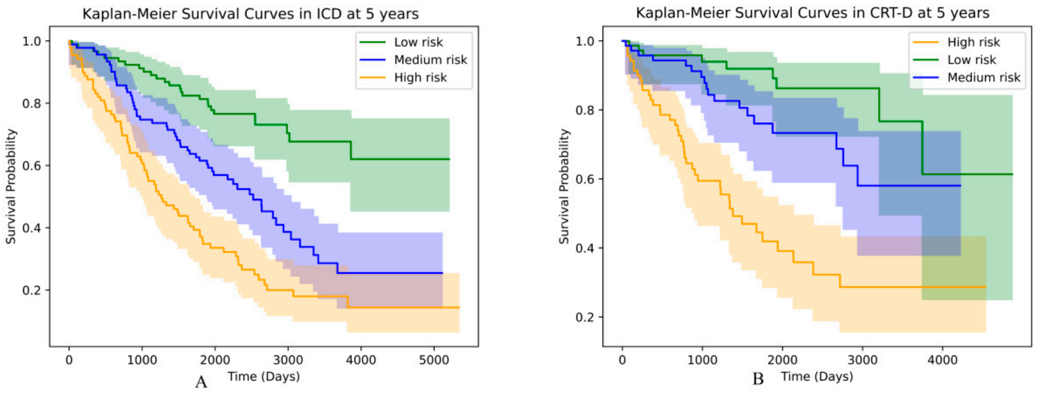

Figure S7. Time-dependent AUC curves for different models in (A) ICD (B) CRT-D

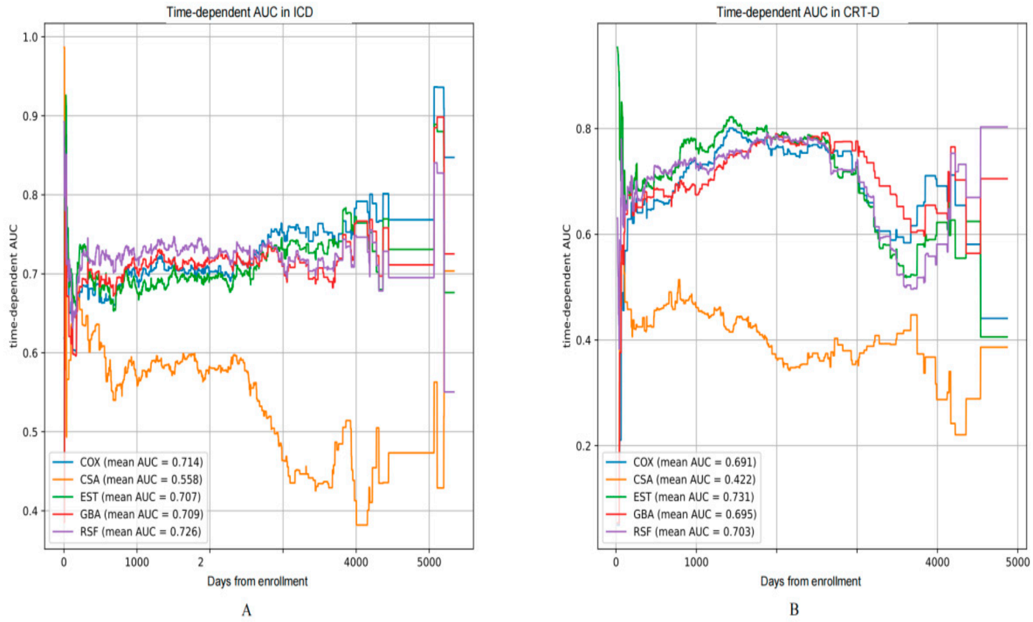

Figure S8. Time-dependent AUC in ischemic and nomischemic CM

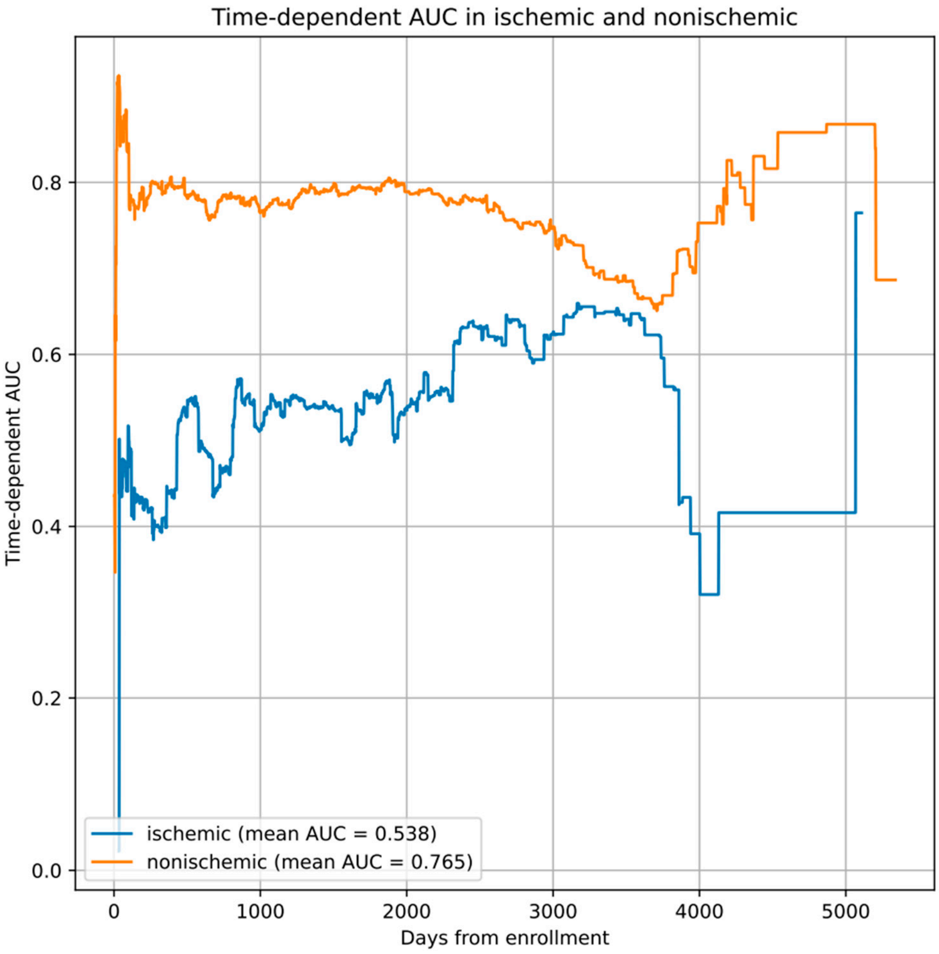

Figure S9. Kaplan－Meier survival curves stratified by the RSF model’s predicted survival probabilities at the 5-year time point. (A) ischemic CM(B) nonischemic CM

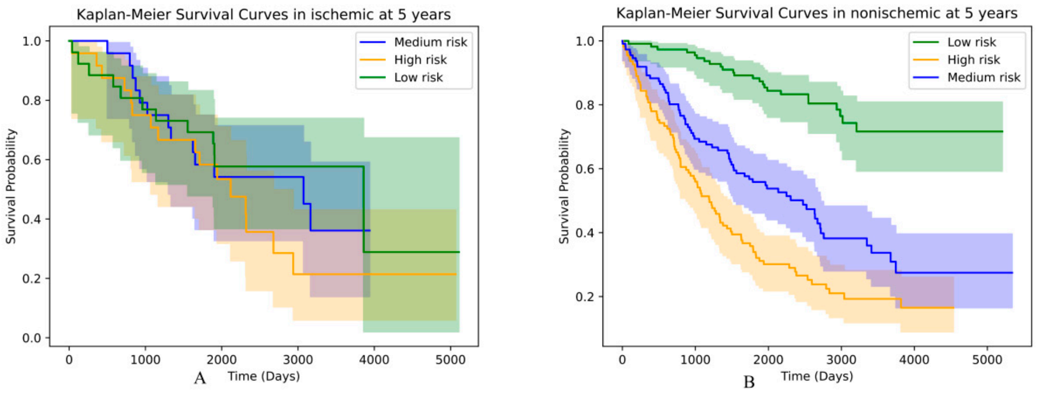

Figure S10. Time-dependent AUC curves for different models in (A) ischemic CM (B) nonischemic CM

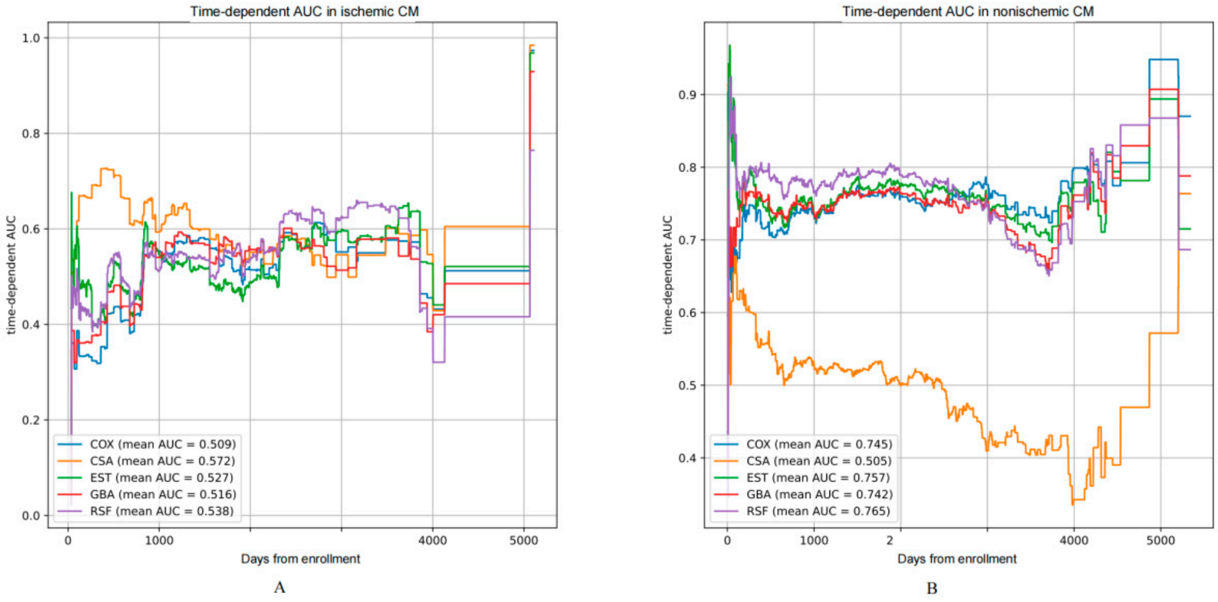

Supplement: Supplementary file 1 [file jcdd-13-00514-s001.zip › jcdd-4540485-supplementary.pdf]
